# Cartilage oligomeric matrix protein specific antibodies are pathogenic

**DOI:** 10.1186/ar4022

**Published:** 2012-08-20

**Authors:** Hui Geng, Kutty Selva Nandakumar, Anna Pramhed, Anders Aspberg, Ragnar Mattsson, Rikard Holmdahl

**Affiliations:** 1 Hubei Key Laboratory of Genetic Regulation and Integrative Biology, College of Life Sciences, Central China Normal University, Wuluo Road 152, Wuhan, 430079, China; 2 Division of Medical Inflammation Research, Department of Medical Biochemistry and Biophysics, Karolinska Institute, Scheeles väg 2, Stockholm, 17177, Sweden; 3 Department of Biology, Cell and developmental biology, University of Copenhagen, Ole Maaløes Vej 5, Copenhagen, DK-2200, Denmark; 4 Department of Experimental Medical Sciences, Biomedical Center, Lund University, Sölvegatan 19, Lund, SE-22184, Sweden

## Abstract

**Introduction:**

Cartilage oligomeric matrix protein (COMP) is a major non-collagenous component of cartilage. Earlier, we developed a new mouse model for rheumatoid arthritis using COMP. This study was undertaken to investigate the epitope specificity and immunopathogenicity of COMP-specific monoclonal antibodies (mAbs).

**Methods:**

B cell immunodominant regions on the COMP molecule were measured with a novel enzyme-linked immunosorbent assay using mammalian expressed full-length mouse COMP as well as a panel of recombinant mouse COMP fragments. 18 mAbs specific to COMP were generated and the pathogenicity of mAbs was investigated by passive transfer experiments.

**Results:**

B cell immunodominant epitopes were localized within 4 antigenic domains of the COMP but with preferential response to the epidermal growth factor (EGF)-like domain. Some of our anti-COMP mAbs showed interactions with the native form of COMP, which is present in cartilage and synovium. Passive transfer of COMP-specific mAbs enhanced arthritis when co-administrated with a sub-arthritogenic dose of a mAb specific to collagen type II. Interestingly, we found that a combination of 5 COMP mAbs was capable of inducing arthritis in naive mice.

**Conclusions:**

We have identified the specificities of mAbs to COMP and their contribution to the development of arthritis. These findings will further improve our understanding of the autoantibody mediated immunopathologies occurring widely in rheumatoid arthritis (RA), as well as in other autoimmune disorders.

## Introduction

Rheumatoid arthritis (RA) is believed to be an autoimmune disease involving an antibody response to citrullinated proteins (ACPA) [1,2] and Ig-Fc (rheumatoid factor, RF) [3]. In many patients with established disease, an antibody response to joint cartilage may also appear [4]. Both antibodies to native triple helical collagen type II (CII) and ACPA recognizing citrullinated CII have been shown to induce arthritis in mice [5-8]. Clinical trials of B cell depletion treatment using rituximab, an anti-CD20 monoclonal antibody, which targets and deletes CD20-expressing B cells, achieved promising clinical outcomes in RA patients [9,10]. These findings in both patients and animal models highlight the role of antibodies in RA.

Cartilage oligomeric matrix protein (COMP) is a major glycoprotein in the extracellular matrix (ECM) of cartilage and synovium [11]. Its biological importance in cartilage was identified in the assembly of the ECM, where COMP interacts with fibrillar collagen types I and II and the FACIT collagen type IX [12,13]. Mutations in the COMP gene have been linked to two human skeletal dysplasias, pseudoachondroplasia (PSACH) and multiple epiphyseal dysplasia (MED) [14,15].

We have developed a new mouse model for rheumatoid arthritis using COMP, where immunization with rat COMP is associated with development of autoimmune arthritis by cross-reactive immune response to autologous mouse COMP [16,17]. The pathology of arthritis induced by COMP immunization shows similarities with human RA having synovial hyperplasia, increased synovial volume, cellular infiltrates, and the unique feature of a chronic relapsing disease phase with a female preponderance. As in RA, the development of arthritis induced by COMP is associated with certain major histocompatibility complex (MHC) haplotypes, indicating that the COMP-induced arthritis (COMPIA) model is dependent on T cell recognition of related peptides presented by appropriate MHC molecules. However, T-cell reactivity alone could not explain the disease immunopathology in COMPIA. COMP-immunized mice have been shown to develop a strong and specific IgG response to COMP, and analysis of blood cell populations in arthritic mice showed an increase in B cells, CD4^+ ^T cells but not cytotoxic CD8^+ ^T cells. Furthermore, arthritis can be transferred from arthritic mice to healthy recipients with affinity purified COMP-specific polyclonal antibodies [17]. It has been earlier reported that anti-COMP antibodies exist in RA synovium and serum, which possibly reflects joint local B cell immune responses toward this cartilage- and tendon-restricted antigen [18].

**Figure 1:**
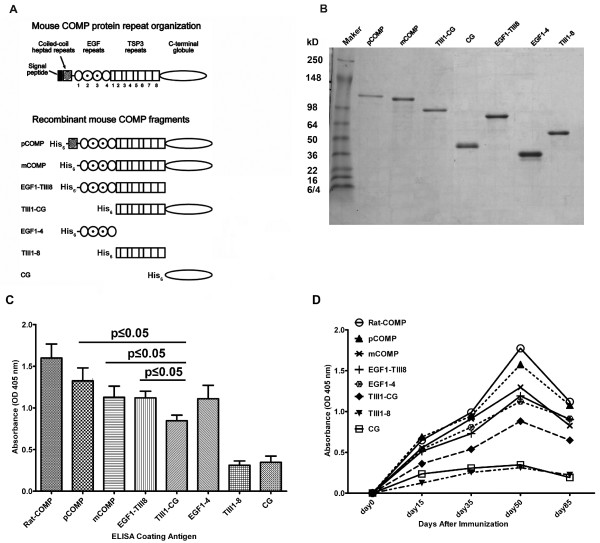
**Relative level and kinetics of the antibody response to each truncated cartilage oligomeric matrix protein (COMP) fragment**. **(A) **Graphical representation of the domain composition of the recombinant mouse COMP proteins used in this study. pCOMP: pentameric COMP; mCOMP: monomeric COMP; EGF: epidermal growth factor; TIII: thrombospondin type 3; CG: C-terminal globular domain. **(B) **Medium was collected from each transfected cell line and purified using Ni-NTA affinity resin followed by a concentration step using ion-exchange chromatography as described in Materials and methods, and 10 μl of the purified proteins were subjected to 4 to 10% gradient SDS-PAGE under reducing conditions. **(C) **Relative level of antibody response to different recombinant COMP fragments. Full length COMP (pCOMP) and COMP fragments (10 μg/ml in 100 μl) were tested by ELISA for COMP autoantibody response. Serum samples from COMP^+/+^Ncf1*/*mice were taken at day 50 after immunization with rat COMP and diluted 1:1000 for the assay. Background levels were determined for each test using antigen, coated, but without primary antibodies in the wells. Results are expressed as absorbance reading at 405 nm and represent the mean and SEM of 13 mice. The lower binding to the TIII1-CG fragment compared to pCOMP, mCOMP or EGF1-TIII8 was confirmed to be statistically significant (*P *≤ 0.05). **(D) **Kinetics of antibody response to rat COMP, full length mouse COMP (pCOMP) and mouse COMP fragments. Serum samples from COMP^+/+^Ncf1*/* mice were taken at days 15, 30, 50 and 85 after immunization with rat COMP and were subjected to ELISA. Sera were diluted 1:1000 for the assays. Data represent the mean values from 13 mice.

COMP is the fifth member of the thrombospondin (TSP) protein family, which includes TSP-1, TSP-2, TSP-3, TSP-4 and COMP/TSP-5. COMP is a homopentamer and each of its subunits consists of an N-terminal coiled-coil oligomerization domain, four EGF-like repeats, eight calcium-binding type 3 (TSP3) repeats and a C-terminal globular domain (Figure 1). To fully understand the immunological events in COMP induced arthritis, it is necessary to identify the immunodominant region of B cell reactivity and demonstrate the contribution of antibodies in arthritis pathology. To identify the domains of the COMP molecule that are recognized by antibodies, we produced mammalian-expressed full-length mouse COMP and a panel of overlapping recombinant mouse COMP fragments. Furthermore, we developed 21 monoclonal antibodies directed to rat COMP, 18 mAbs reactive with rat COMP cross-reacted with mouse COMP. Here, we demonstrate the specificity of polyclonal antibodies and monoclonal antibodies for different domains of the COMP molecule. COMP-specific mAbs interact with native COMP in cartilage and synovium, as evidenced by the binding of biotinylated mAbs *in vivo*. Passive transfer of selected anti-COMP mAbs- enhanced arthritis when co-administered with a sub-arthritogenic dose of a mAb that specifically recognizes the J1 epitope of the CII molecule [19]. A combination of five selected COMP mAbs, without anti-CII mAb, was also effective to induce arthritis in naïve mice, although with low severity of arthritis.

## Materials and methods

### Animals

COMP-deficient 129/Sv mice [20] were backcrossed for 10 generations to B10.Q mice to introduce the A^q ^allele in the MHC locus. B10.Q mice with a mutated *Ncf1 (Ncf1^m1J ^*denoted as Ncf1*) have been described previously [21]. To obtain mice with COMP-deficiency and *Ncf1 *mutation (COMP-/-Ncf1^*/*^), COMP-deficient B10.Q mice were intercrossed with *Ncf1 *mutant mice to get COMP-deficient and *Ncf1 *mutated heterozygous mice. These heterozygous mice were intercrossed and the offspring were investigated for COMP deficiency and *Ncf1 *mutation by PCR and DNA sequence respectively [21,22]. For passive transfer experiments (BALB/c × B10.Q) F1 male mice (known as QB) were used; these showed higher sensitivity to antibody-induced arthritis [7]. All the mice were kept and bred in a climate-controlled environment with a 12-h light/dark cycle. Mice were housed in polystyrene cages containing wood shavings and fed standard rodent chow and water *ad libitum *in the animal house of Medical Inflammation Research, Sweden. All the experiments were performed using age-matched mice between 8 and 10 weeks and blinded to the investigator. Malmö/Lund and Stockholm ethical committees approved all the experiments.

### Production of recombinant COMP proteins

The corrected cDNA for mouse COMP [22] served as the template for PCR amplification of the following recombinant COMP fragments (Figure 1). In addition to full-length his-tagged mouse COMP (pCOMP, nucleotide 82-2289), the fragments produced were; (1) mCOMP, monomeric COMP, that is, only lacking the coiled-coil repeats, nucleotide 238-2289; (2) EGF1-TIII8, EGF-like repeats and TSP3 repeats, nucleotide 238-1569; (3) TIII1-CG, TSP3 repeats and C-terminal globular domain, nucleotide 820-2289; (4) EGF1-4, EGF-like repeats, nucleotide 238-819; (5) TIII1-8, TSP3 repeats, nucleotide 820-1569, and (6) CG, C-terminal globular domain, nucleotide 1570-2289 [NM_016685.2, GenBank]. The mouse COMP recombinant fragment cDNAs were amplified using 5'-phosphorylated upstream primers in combination with downstream primers adding a stop codon followed by an Nhe I site. The PCR products were digested with Nhe I and inserted into the Pvu II and Nhe I sites of the expression vector pCEP4-TAGZyme. This plasmid was constructed by inserting the sequence 5'-GGTACCGCCTGCCGCCTGCCT GCCTGCCACTGAGGGTTCCCAGCACCATGAGGGCCTGGATCTTCTTTCTCCTTTGCCTGGCCGGGAGGGCTCTGGCAGCCCGACATCACCATCACCATCACCAGCTGAAGCTT-3' between the Kpn I and HinDIII sites of plasmid pCEP4, yielding an expression vector with a BM40 signal peptide followed by a hexahistidine tag compatible with the TAGZyme removal system (Qiagen, Copenhagen, Denmark). All the PCR-generated fragments and their cloning sites were confirmed by nucleotide sequencing.

The resulting recombinant COMP plasmids were transfected into 293-c18 cells (ATCC CRL-10852) using the FuGene6 reagent (Roche, Basel, Switzerland ) and transfected cells were selected with hygromycin and grown to confluence in Dulbecco's modified Eagle's medium with 10% fetal calf serum (Gibco, Life Technologies Inc, Camarillo, CA, USA). Secretion of recombinant proteins into the medium was confirmed by SDS-PAGE of samples from transfected and non-transfected cells. Serum-free culture medium was harvested, and his-tagged recombinant mouse COMP proteins were purified using Ni^2+^-metal chelating and MonoQ ion exchange chromatography. Purity and size of all the proteins were assessed by SDS-PAGE and colloidal Coomassie Brilliant Blue G250 staining with or without prior reduction of disulfide bonds.

### Rat COMP purification

Purification of COMP from the Swarm rat chondrosarcoma was performed as previously described [17]. Briefly, 50 g rat chondrosarcoma was homogenized and centrifuged, the pellet was extracted in 150 m*M *NaCl, 50 m*M *Tris, 10 m*M *EDTA, pH 7.4, supernatants were harvested, and COMP was purified using DEAE-Sepharose fast-flow media column (GE Healthcare, Uppsala, Sweden), COMP-containing fractions were evaluated by SDS-PAGE. Pooled fractions were then exchanged to 150 mM NaCl, 10 mM Tris, and 2 mM EDTA, pH 7.4 buffer. Pooled fractions were concentrated and negatively purified using HiTrap heparin-Sepharose column, HiTrap Q Sepharose column, heparin column (GE Healthcare, Uppsala, Sweden) and Q column. The purity and size of COMP fractions were assessed by SDS-PAGE under reducing and non-reducing conditions.

### Synthetic peptides

Sequence alignment identified 15 amino acid differences between the mature peptide sequences of rat and mouse COMP. The following peptides, corresponding to the rat COMP sequence and containing the amino acids differing from mouse COMP (underlined), were synthesized using methods previously described in detail [8]. P1: DVRELLRHRVKEITFLK (in coiled-coil repeats); P2: PGLSVRPVALCAPGSCF (in EGF1); P3: SCFPGVVCTETATGARC (in EGF1); P4: GVGLTFAKTNKQVCTDI (in EGF2); P5: QPGFVGDQRSGCQRRGQ (in EGF3); P6: PSPCHEKADCILERDGSRS (in EGF4); P7: GQEDVDRDRIGDACDPD (in TIII2); P8: NPDQRNSDKDKWGDAC D (in TIII3); P9: DACDNCRSQKNDDQKDT (in TIII4); P10: NDDQKDTDRDGQG DACDDDI (in TIII4); P11: DTIPEDYERHRLRRA (C-terminal peptide). Peptide identities were confirmed by mass spectrometry and fast protein liquid chromatography was used to maintain purity at > 98% (Hermann GbR Synthetische Biomoleküle, Freiburg, Germany).

### Generation of COMP-specific B cell hybridomas

Hybridomas producing anti-COMP mAbs were generated as described earlier [23]. Briefly, COMP^-/-^Ncf1^*/* ^and COMP^+/+^Ncf1^*/* ^mice were immunized at the base of the tail with 100 μg of rat COMP emulsified in complete Freund's adjuvant (CFA; Difco, Detroit, MI, USA), followed by an i.p. booster with 50 μg of rat COMP in PBS. Three to five days after the booster dose, spleen or inguinal lymph cells were fused with SP2/0 or NSO-bcl2 myeloma cell lines. Two weeks after the fusion, all the primary plates were screened for antibody production by ELISA using rat COMP. Cells from positive wells were sub-cloned 5 times by limiting dilution and expanded for further use. Monoclonal antibodies were purified from the supernatants by affinity chromatography using protein G column (GammaBind plus Sepharose; GE Healthcare, Uppsala, Sweden) and bound IgG was eluted in 0.1 M glycine (pH 2.8) and neutralized with one-sixth volume of 1 M Tris-HCl (pH 8.5). The eluted fractions were dialyzed against PBS buffer extensively. Monoclonal antibodies were quantified by measuring absorbance at 280 nm and the purity of the antibodies was assessed by SDS-PAGE. The endotoxin content in all mAbs preparations was found to be below detection limit (less than 1 EU/mg) as analyzed with the *Limulus *amebocyte lysate (Pyrochrome) method (Cape Cod Inc., Falmouth, MA, USA).

### Antibody measurements

Enzyme immunoassays were performed as described earlier [22]. Briefly, 96-well flat-bottom ELISA plates (Nunc MaxiSorp; eBioscience, San Diego, CA, USA) were coated with 10 μg/ml of recombinant COMP proteins, or with 10 μg/ml synthetic COMP peptide in PBS at 4°C and left overnight. Plates were blocked with 1% bovine serum albumin in PBS containing Tween, incubated with the supernatants from hybridomas or sera, washed, and again incubated with goat anti-mouse IgG peroxidase-conjugated antibodies (Jackson Immuno-Research, West Grove, PA, USA) and ABTS tablets (Roche) as substrate. For isotype-specific assessment, biotinylated anti-IgM, -IgG1, -IgG2a, -IgG2b, or -IgG3 reagents (Jackson Immuno-Research, West Grove, PA, USA) were used for detection.

### Passive transfer of antibodies and evaluation of arthritis

The anti-CII mAb (M2139) binding to the MPGERGAAGIAGPK epitope of the CB10 fragment (aa 551-564) of the CII molecule used in this study was produced as described earlier [6,19]. The cocktail of M2139 and COMP mAbs and the combination of COMP mAbs were prepared by mixing equal concentrations of each of the sterile-filtered antibody solution to achieve a final amount of 9 mg. On day 5 (or day 7), all the mice received 25 μg/mouse lipopolysaccharide (LPS) from *Escherichia coli *serotype O26:B6 (Sigma-Aldrich, Saint Louis, MO, USA) intraperitoneally to enhance the severity of the arthritis developed. Arthritis development was monitored daily in a blinded fashion for 16 days using an extended scoring protocol [24]. Briefly, clinical arthritis is defined as swelling and redness in the joint and was scored as follows: 1 point was given for each swollen or red toe, 1 point for each swollen joint (metatarsal phalangeal joints, metacarpal phalangeal joints, proximal interphalangeal joints, and distal interphalangeal joints), and 5 points for a swollen ankle (maximum score per limb was 15 and maximum score per mouse was 60).

### Histology

For histological assessments, knee joint and paws from adult mice were dissected, decalcified, dehydrated, and then paraffin-embedded, as described previously [23]. Sections (5 μm) were stained with hematoxylin/eosin or toluidine blue. For analysis of anti-COMP mAb reactivity with joint tissue *in vivo*, neonatal mice were injected with 100 μg biotinylated mAbs against COMP intraperitoneally. After 24 h, knee joints were snap frozen in isopentane on dry ice and stored at -70°C. Joint sections (7 μm) were fixed in 4% paraformaldehyde for 5 minutes, rinsed in PBS, incubated with Extravidin peroxidase (Sigma-Aldrich, Saint Louis, MO, USA) for 30 minutes and developed with diaminobenzidine (DAB Kit; Dako, Copenhagen, Denmark) for 8 to 9 minutes. To assess direct binding of anti-COMP mAbs to the tissue sections, limbs from naïve neonatal mice were harvested and snap frozen, cryo-sectioned, and sections of 7 μm were subjected to 5 μg/ml biotinylated mAbs against COMP for 40 minutes. Extravidin peroxidase and DAB were used for the detecting system.

### Statistical analyses

Quantitative data are expressed as mean ± standard error of the mean (SEM). Arthritis incidence was analyzed using chi-square or Fisher's exact test (SAS Institute; Cary, NC, USA) and comparison of severity was performed using the non-parametric Mann-Whitney *U*-test. Analysis of variance (ANOVA) was used to analyze polyclonal Ab response statistics. Differences with *P*-values less than 0.05 were considered statistically significant.

## Results

### Specificity of polyclonal antibodies to COMP

To perform epitope mapping of COMP, we expressed recombinant his-tagged full-length mouse COMP and a panel of overlapping COMP fragments in HEK 293-c18 cells (Figure 1A). All the recombinant proteins were purified using affinity and ion exchange chromatographic methods. For each recombinant protein, the expected apparent molecular weight and a purity of > 95% was verified by SDS-PAGE analysis (Figure 1B).

To ascertain whether antibodies show reactivity towards individual full-length and truncated form of COMP proteins, microtiter plates were coated with either recombinant COMP molecules or COMP purified from rat chondrosarcoma, and used in enzyme immunoassays with sera obtained at 50 days after primary immunization. As shown in Figure 1C, reaction with recombinant COMP proteins was in the following order: pCOMP > mCOMP approximately = EGF1-TIII8 approximately = EGF1-4 > TIII1-CG > CG approximately = TIII1-8. Notably, of the single domain fragments, reactivity to EGF1-4 is considerably higher than to TIII1-8 or CG. However, in the native fold, the TSP3 repeats (TIII1-8) are wrapped around the C-terminal globule (CG) making direct comparison complicated. Nevertheless, the lower reactivity to the TIII1-CG fragment (the complete TSP3 and C-terminal globule fragment), compared to all the fragments containing the EGF repeats (pCOMP, mCOMP, EGF1-TIII8 and EGF1-4) underlines an important role of the EGF repeats in the immune response.

In COMP^+/+^Ncf1^*/* ^mice, the incidence of arthritis was 80% with the day of disease onset at 36 to 38 days [17]. Since cross-reactions with autologous COMP appear to be of importance for the development of arthritis, we analyzed the antibody reactivity to the autologous mouse recombinant full-length COMP and COMP fragments. Immunized mice produced a rapid and strong IgG response as early as 14 days after immunization (Figure 1D). Antibody reactivity to both rat and mouse COMP showed a definite trend of increasing serum titres, from day 14 onwards with a peak response around day 50. Analysis of the kinetics of antibody response to each of the recombinant COMP fragments confirmed the important contribution of EGF repeats to the overall immune response. Antibody levels to the EGF1-4 fragment were higher than observed for TIII1-8, CG and TIII1-CG fragments at all time points tested in our experiments.

### Generation and characterization of anti-COMP mAbs

To obtain a broad set of COMP-specific mAbs, we generated a panel of mAbs using COMP-deficient mice and COMP-wild type mice with *Ncf1 *mutation immunized with the native form of rat COMP during secondary immune response. A total of 21 IgG mAbs from four separate fusions were developed and sub-cloned to obtain stable single clones. None of the COMP antibodies cross-reacted to CII, which might be present as a minor contaminant with the rat COMP during protein preparation from the Swarm rat chondrosarcoma. Isotype analysis revealed that all of the COMP mAbs generated were of IgG isotype, containing IgG1, IgG2a, and IgG2b subtypes, indicating that they were indeed produced by T cell-dependent follicular B cells (Table 1). Since we were interested in antibodies capable of inducing arthritis, we screened each antibody for its ability to bind autologous mouse COMP, a total of 18 mAbs reactive with rat COMP cross-reacted with mouse COMP (Table 1). Five of these 18 mAbs were cross-reactive to human COMP, which suggests that these epitopes on the COMP molecule are evolutionarily conserved.

**Table 1:** Summary of mAb specificities

Clones	Mice	Isotypes	Rat COMP	Mouse COMP	Human COMP	Binding sites
1A12	COMP^-/-^	IgG1	++	++	_	EGF-like domain
1B8	COMP^-/-^	IgG1	+	+	_	TSP3 domain
1D10	COMP^-/-^	IgG1	++	++	++	C-terminal globular domain
2D3	COMP^-/-^	IgG1	+++	+++	+++	coiled-coil domain or pentameric COMP
1C1	COMP^-/-^	IgG2b	+	++	_	EGF-like domain
3C7	COMP^-/-^	IgG2b	+	_	_	ND
3F8	COMP^-/-^	IgG2b	++	++	_	EGF-like domain
4A8	COMP^-/-^	IgG2b	++	++	_	C-terminal globular domain
8E3	COMP^-/-^	IgG1	++	+	_	EGF-like domain
12H8	COMP^+/+^	IgG1	+++	+++	_	EGF-like domain
15A11	COMP^+/+^	IgG1	+++	+++	+++	EGF-like domain
4D12	COMP^-/-^	IgG2b	+	_	_	EGF-like domain
3F11	COMP^-/-^	IgG2a	++	+	_	EGF-like domain
16B5	COMP^+/+^	IgG1	+++	+++	+++	coiled-coil domain or pentamer COMP
18E7	COMP^-/-^	IgG2b	+	+	_	EGF-like domain
5E8	COMP^-/-^	IgG2b	+	_	_	ND
6H4	COMP^-/-^	IgG2b	++	++	+	EGF-like domain
29C3	COMP^-/-^	IgG1	+	+++	_	EGF-like domain
30G5	COMP^-/-^	IgG2b	++	++	_	C-terminal globular domain
23A8	COMP^-/-^	IgG1	++	+++	_	EGF-like domain
24F7	COMP^-/-^	IgG2b	++	++	_	EGF-like domain

Grading of values obtained with mAbs using culture supernatants. Scale for optical density (OD) values: -, < 3 times standard error of the mean (SEM) of negative control; +, > 3 times SEM of negative control; ++, > 0.5 OD; +++, > 1.0 OD; COMP, cartilage oligomeric matrix protein; EGF, epithelial growth factor; ND, not determined.

**Figure 2:**
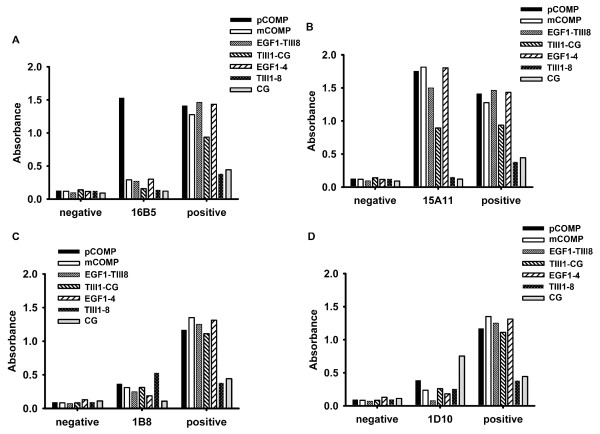
**Epitope mapping of 4 representative COMP-specific mAbs by using full-length cartilage oligomeric matrix protein (COMP) and recombinant fragments**. B cell hybridomas were produced from COMP^+/+^Ncf1^*/* ^or COMP^-/-^Ncf1^*/* ^immunized with rat COMP. Monoclonal antibodies were obtained from culture supernatants after 14 days and tested by ELISA. Plates were individually coated with 10 μg/ml of recombinant full-length mouse COMP or COMP fragments. Serum samples from naïve mice served as negative controls and serum samples from COMP immunized mice were used as positive controls.

**Figure 3:**
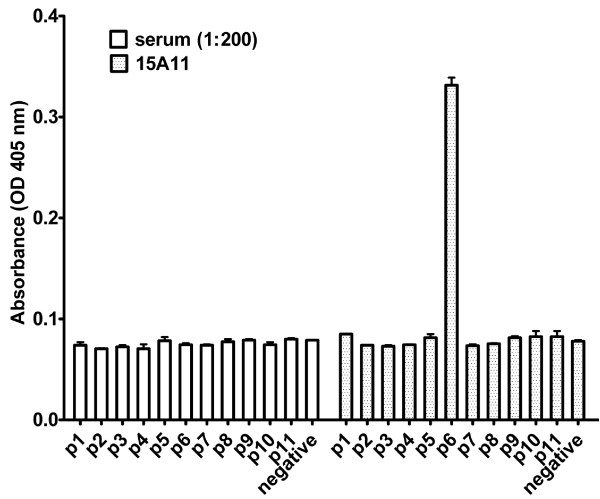
**Fine mapping of selected cartilage oligomeric matrix protein (COMP)-specific mAbs**. To identify possible linear peptide epitopes for anti-COMP mAbs, synthetic peptides from rat COMP were produced. Each peptide spans amino acid residues differently in the rat and mouse sequence. Antibodies were obtained from culture supernatants after 14 days and tested by ELISA in wells individually coated with 10 μg/ml of synthetic peptide. Pooled sera from COMP-immunized mice were used as the positive control. Monoclonal Ab 15A11 bound peptide 6, which is derived from EGF repeat 4.

For the detection of mAbs binding to different regions of COMP molecule, we performed ELISA with the recombinant COMP fragments. The binding properties of 4 representative mAbs to the truncated COMP fragments are shown in Figure 2. The 16B5 clone produced a specific signal only with pCOMP, which is a full length COMP molecule, but produced no signal with any of the other COMP fragments. Hence, we deduced that 16B5 binding site is either located at the N-terminal coiled-coil pentamerization domain or a conformational discontinuous epitope of pentameric COMP. The 15A11 clone represents mAb with a binding site located at the EGF-like repeat domain, which produced a specific signal with pCOMP, mCOMP, EGF1-TIII8 and EGF1-4 fragments but produced no signal with TIII1-8 and CG fragments. This antibody does, however, also show reactivity to the TIII1-CG fragment, although producing a lower signal. The 1B8 clone represents mAb having a binding site located at the TSP3 domain, which produced a specific signal with pCOMP, mCOMP, EGF1-TIII8, TIII1-CG and TIII1-8 fragments, but produced no signal with EGF1-4 and CG fragments. The 1D10 clone represents mAb binding to epitopes that are located at the C-terminal globular domain, which produced a strong specific signal with the CG fragment, bound the CG containing fragments pCOMP, mCOMP, TIII1-CG, but produced little or no signal with the EGF1-4, EGF1-TIII8 and TIII1-8 fragments. This mapping strategy using the recombinant COMP fragments was applied on each of the 18 mouse-specific mAbs. The mAbs binding sites were thereby narrowed down to one of the COMP domains. Among these 18 mAbs, 12 reacted with the EGF-like domain. Alignment of the rat and mouse COMP sequences revealed 15 amino acid differences between the mature peptide sequences. Considering that these differences could result in a strong immune response, we produced 11 synthetic peptides corresponding to the rat COMP sequence, each spanning one or two variant residues. To fine map the anti-COMP mAb epitopes, we performed ELISA on the 11 synthetic peptides with the hope that some of the mAbs would react to a linear peptide structure. As shown in Figure 3, the peptide PSPCHEKADCILERDGSRS, which locates at EGF-repeat 4, was found to bind the 15A11 mAb. None of the other mAbs tested (16B5, 12H8, 1B8, 2D3, 3F8, 6H4) showed binding to any of the peptides tested.

### Monoclonal antibodies specific for COMP bind cartilage

**Figure 4:**
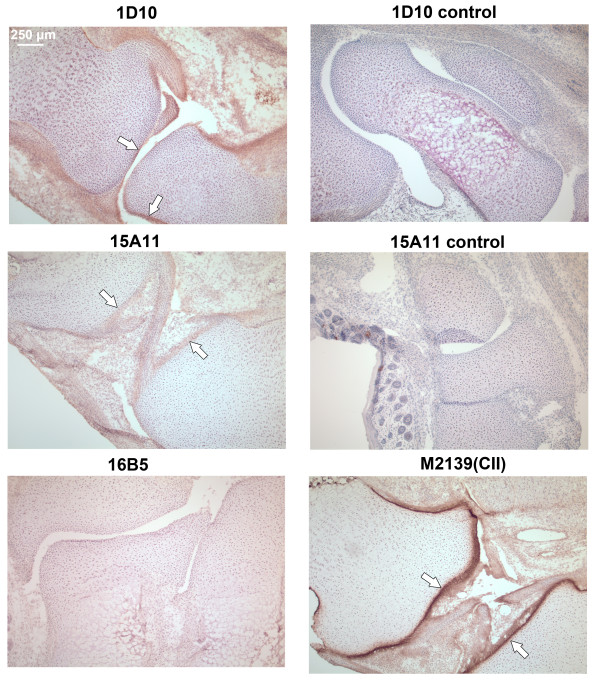
**Immunohistochemical analysis of cartilage oligomeric matrix protein (COMP)-specific mAbs binding to joint cartilage *in vivo***. COMP specific-mAbs were purified, biotinylated and injected into neonatal COMP^+/+ ^mice (100 μg of antibody in 100 μl of PBS/mice) and limbs were collected after 24 h. Seven μm cryo-sections were stained with DAB using extravidin-peroxidase as the detection system. Anti-CII monoclonal antibody (M2139)-injected animals served as positive controls, anti-COMP mAb injected COMP^-/- ^mice were used as negative controls. Positive mAb binding to joint surfaces is marked by arrows.

To test whether the mAbs bind with native COMP in cartilage, COMP mAbs were biotinylated and injected separately into neonatal COMP^+/+ ^mice and the presence in joints of biotinylated mAbs after 24 h was analyzed using cryo-sections of whole paws. The mAbs demonstrated specific binding to cartilage and synovial tissue, with different affinity to the joint cartilage tissue (Figure 4). The 1D10 and 15A11 mAbs gave detectable staining, showing accumulation on the lining of the normal articular cavity, most visibly along the cartilage surface and synovial tissue. In contrast, mAb 16B5 showed no appreciable staining. The specificity for COMP was verified by performing the same experiment in neonatal COMP^-/- ^mice, where no joint staining was observed (Figure 4).

Cartilage tissue is a dense ECM with a very high concentration of proteoglycans, suggesting that antibody penetration into the tissue would be limited. In agreement with this, the anti-collagen II (CII) mAb M2139 staining was restricted to the cartilage surface (Figure 4). The 1D10 and 15A11 mAbs stainings, although weaker than the CII staining, show the same pattern, with additional staining of the synovium and bone.

**Figure 5:**
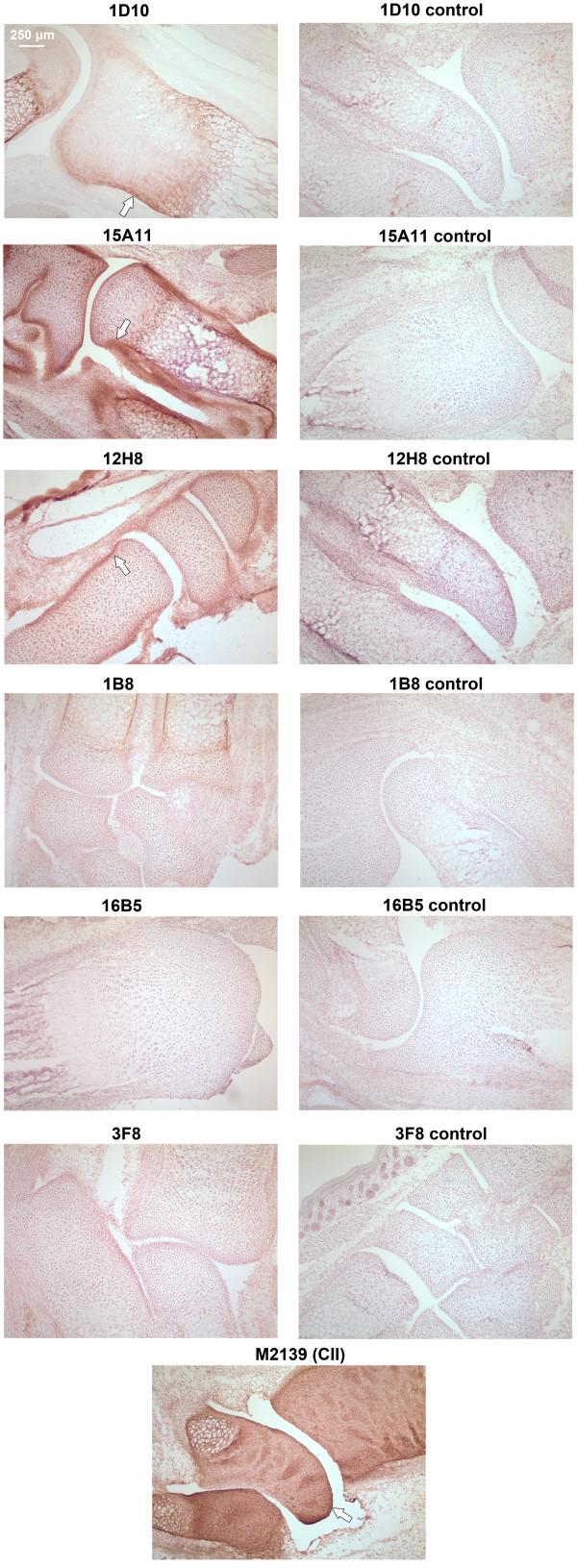
**Immunohistochemical analysis of cartilage oligomeric matrix protein (COMP)-specific mAbs binding to cartilage *in vitro***. Immunohistochemistry was performed using 7 μm cryo-sections from naive neonatal mouse limbs, using 5 μg/ml of biotinylated COMP-specific mAbs. Binding was detected by extravidin-peroxidase and DAB substrate. Sections from COMP^+/+ ^mouse limbs stained with 12H8, 15A11, 1D10, 3F8, 1B8, 16B5 are depicted in the pictures. Sections from COMP^-/- ^mouse limbs stained with 12H8, 15A11, 1D10, 3F8, 1B8, 16B5 were used as negative controls. Anti-collagen type II (CII) monoclonal M2139 served as the positive control. The positive monoclonal staining on cartilage and tendon is indicated with an arrow.

To verify the *in vitro *binding capacity of mAbs to cartilage, joint sections from untreated neonatal wild-type or COMP-/- mice were incubated with the individual biotinylated mAbs and binding of the antibody detected by indirect immunohistochemistry (Figure 5). As expected, mAb M2139 in this case gave uniform CII staining throughout the cartilage from wild-type mice. This was also the case for the three COMP mAbs that showed clear cartilage staining in wild-type mice (1D10, 15A11 and 12H8).

### COMP-specific monoclonal antibodies induce and enhance severe acute arthritis in naïve mice

**Figure 6:**
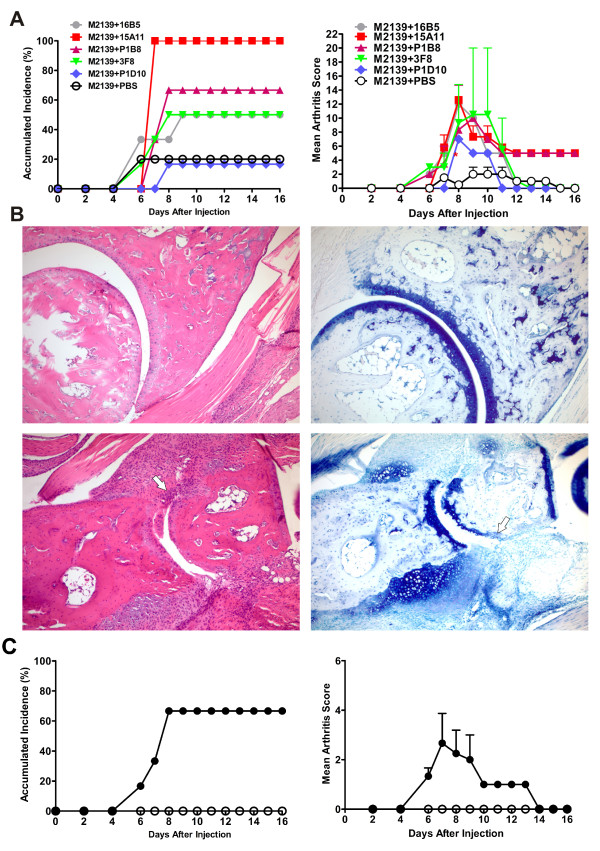
**Cartilage oligomeric matrix protein (COMP)-specific mAbs mediate arthritis**. **(A) **Accumulated incidence and severity of arthritis. Two-month-old naïve male QB F1 mice were injected intravenously (i.v.) with 9 mg of an equal combination of M2139 with a single COMP-mAb. All the mice received lipopolysaccharide (25 μg per mouse) intraperitoneally on day 5 and were scored for arthritis up to 16 days. The error bars in the severity graph indicate standard error of the mean (SEM), *n *= 6 per combination group. **(B) **Histology of tarsal joint sections. Paws of QB F1 mice on day 16 after the antibody transfer were collected, fixed, decalcified, sectioned and stained with hematoxylin/eosin (left panel) or toluidine blue (right panel). Top panel, M2139 plus PBS-injected control mice; Bottom panel, M2139 plus 15A11-injected mice. Note thickened synovial lining, inflammatory cellular infiltrate extending over the cartilage surface and erosions of cartilage and bone in animals injected with M2139 plus 15A11 (bottom). Results shown are representative of those obtained from three to four mice in each group. Infiltration cells, glycosaminoglycan loss and joint surface erosions are indicated with arrows. **(C) **Accumulated incidence and severity of arthritis. Two-month-old naïve male QB F1 mice were injected i.v. with 9 mg of an equal combination of 1B8, 1D10, 3F8, 15A11 and 16B5 anti-COMP monoclonal antibodies (filled circles). The same amount of a combination of isotype-matched control antibodies (L243 + G11) was injected into a separate group of animals (open circles). Results shown are pooled values from two similar experiments with balanced groups. The error bars in (**A**) and (**B**) indicate the SEM.

To investigate the pathogenic potential of COMP-specific mAbs, (BALB/c × B10.Q) F1 mice were first injected with single COMP-specific mAbs in combination with a sub-arthritogenic dose of M2139, a CII specific IgG2b mAb. This experimental design was chosen because most of the COMP-specific mAbs are of the poorly complement-fixing IgG1 isotype, and complement activation is one of the critical factors for development of arthritis [25,26]. We have earlier reported that 4.5 mg of M2139 induced no visible arthritis [7]. This dose was therefore chosen to use in combination with different COMP-specific mAbs, to test whether these mAbs could contribute to tissue injury by enhancing the subclinical disease established by the CII-specific M2139 antibody alone. Indeed, combination of single COMP-specific mAbs with a sub-optimal dose of M2139 mAb did induce arthritis (Figure 6A). Among the different combinations tested, the 15A11 plus M2139, and the 3F8 plus M2139 showed the most efficient arthritogenic activity. In the 15A11 plus M2139 group, all six mice presented aggressive disease, which may indicate that they were most effective at complementing each other. In the 3F8 plus M2139 group, four out of six mice presented aggressive disease (Figure 6A). To confirm the clinical observations, paws from treated animals were subjected to histopathological examination after the termination of the experiments. In the tissue sections of arthritic mice, we could detect inflammatory infiltration in the synovium and articular cavity, synovial hyperplasia, pannus formation, bone and cartilage erosions, and furthermore proteoglycan loss was clearly evident (Figure 6B).

To further test whether a combination of antibodies to the most dominant epitopes of COMP was arthritogenic, anti-COMP mAbs 1B8, 1D10, 3F8, 15A11 and 16B5, which bind sites located at different domains of COMP, were mixed at equal concentrations and injected intravenously into 2-month-old mice. Clinical symptoms of arthritis developed in four out of six mice characterized by a rapid onset albeit with a low disease severity (Figure 6C). No signs of arthritis were observed in animals injected with control monoclonal antibodies.

## Discussion

COMP is expressed predominantly in cartilage and synovium [11]. An immune response to COMP leads to arthritis [16,17]. Here we clearly show that arthritogenic antibodies are indeed produced after native COMP immunization that are preferentially directed to the EGF region of the COMP molecule, bind cartilage and induce arthritis in naïve mice.

To analyze the epitope specificity of autoantibodies to COMP, we injected mice with COMP and investigated the ensuing polyclonal antibody response at different time points. Furthermore, we generated monoclonal antibodies from the responding B cells to characterize the fine specificity of various COMP antigenic determinants. Interestingly, the polyclonal antibody response was dominated by the reactivity to the EGF-like domain compared to the TSP3 and C-terminal globular domains at all the time points tested in our experiments. Similar to the anti-COMP polyclonal response, the majority of the mAb (12 out of 18) binding sites are located on the EGF-like domain. These results indicate that the EGF-like domain is the highly immune-dominant region in the B cell immune response to COMP. In the present study, we produced full-length mouse COMP, as well as a panel of overlapping COMP fragments in mammalian cells to preserve the three-dimensional structure of the protein. These recombinant COMP molecules allowed us to select mAbs that are specifically binding to conformational COMP epitopes, which are highly dependent on the three-dimensional structure. Although some investigators have proposed that any surface patch of a protein can be an epitope [27,28], in many cases, immunogenic hot spots are focused on restricted parts of the corresponding autoantigens *in vivo *[29,30]. In the present study, COMP B cell immuno-dominant regions were found to be located at the EGF-like domain of the COMP molecule.

Increase in synovial fluid and serum COMP level is used as a bio-marker for cartilage turnover in human joint diseases [31,32]. Several matrix metalloproteinase and ADAMTS (a disintegrin and metalloprotease with thrombospondin motifs) family members such as ADAMTS-4, -7 and -12 are responsible for COMP degradation [33-35]. Di Cesare and colleagues were able to resolve a major reduction-sensitive 150 KD COMP fragment, which may include the pentamerizing domain and some portion of the type-2 repeats and non-reduction-sensitive 67 to 94 kDa fragments which must be C-terminal fragments present in cartilage extracts of patients with RA and osteoarthritis (OA) [36]. The newly generated mAbs, specific in the present study to COMP molecular domains, may prove useful tools to detect such COMP degradation fragments in the synovial fluid and serum. Hence, extensive investigations are needed to test our mAbs for the detection of degradative fragments of COMP in the synovial fluid and serum of arthritic patients.

Earlier, we reported that the affinity-purified COMP-specific polyclonal antibodies can induce arthritis in healthy recipients [17]. Here, we generated, characterized and identified mAbs that can interact with the native conformation of antigen present in the joint cartilage. Indeed, when wild-type and COMP^-/- ^mice, were immunized with COMP, 80% COMP^+/+ ^mice developed severe arthritis, whereas COMP^-/- ^animals exhibited no clinical abnormalities (our unpublished data). These differences in arthritis severity are very likely a result of the absence of COMP as a target for the anti-COMP immune response. In this study, we provide evidence to show that mAbs to defined epitopes on COMP are indeed pathogenic, thereby emphasizing a link to COMP-mediated autoimmunity. Current observations of binding of COMP-specific mAbs directly to the cartilage COMP in its native conformation, and the passive transfer of COMP-specific mAbs leading to arthritis, have important implications for the understanding of the ensuing immune responses against COMP in arthritis. It has been reported that antibodies play a pivotal role in triggering inflammation in arthritis [7,8,37]. It is most likely that the first step in the initial triggering event in this transfer model is the formation of COMP-IgG immune complexes on the cartilage surface or in the synovium. Cartilage COMP served as a target for the autoantibodies and these immobilized immunoglobulins may activate the complement pathways and/or FcγR-bearing cells. Both C5a receptor and FcγRIII have been reported to play co-dominant roles in the CII antibody induced arthritis (CAIA) and glucose-6-phosphate isomerase serum-induced arthritis [38-40]. It has been reported that synovial macrophages and neutrophils act downstream during the process of antibody-mediated joint inflammation [41,42].

In RA patients, joint-specific autoantigens are considered to play an important role in the induction of synovial B cell expansion [43]. A sign of severe RA is the presence of germinal center-like structures in the inflamed synovial tissue, suggesting a locally generated B cell response to tissue specific autoantigens [44]. Furthermore, a potential role of COMP as an arthritogenic target in RA is derived from clinical studies, where COMP fragments [45,46] and antibody response to COMP [47] have been observed in the serum samples and joint fluids. Recent studies also showed that COMP has the potential to activate the alternative complement pathway and can inhibit the classical and lectin pathways [48]. Thus, autoimmunity to COMP could contribute to the pathogenesis of RA in maintaining the active inflammatory processes ongoing in the inflammatory joint. In this respect, antibodies secreted by synovial B cells could induce inflammatory joint damage in RA patients, in similarity with animal experiments.

## Conclusions

We elucidated specificities of autoantibodies to COMP and its contribution to the development of arthritis. These findings will further improve our understanding of the autoantibody-mediated immunopathologies occurring widely in RA as well as in other autoimmune disorders.

## Abbreviations

ACPA: anti-citrullinated protein antibodies; ADAMTS: a disintegrin and metalloprotease with thrombospondin motifs; COMP: cartilage oligomeric matrix protein; COMPIA: COMP-induced arthritis; pCOMP: pentameric COMP; mCOMP: monomeric COMP; CG: C-terminal globular domain; CII: collagen type II; EGF: epidermal growth factor; ELISA: enzyme-linked immunosorbent assay; ECM: extracellular matrix; LPS: lipopolysaccharide; MED: multiple epiphyseal dysplasia; PSACH: pseudoachondroplasia; PBS: phosphate-buffered saline; RA: rheumatoid arthritis; RF: rheumatoid factor; TSP: thrombospondin; TIII: thrombospondin type 3 repeat.

## Competing interests

The authors declare that they have no competing interests.

## Authors' contributions

RH and HG had full access to all of the data in the study and take responsibility for the integrity of the data and the accuracy of the data analysis. RH, AA and RM designed the study. Experiments were performed by HG, KSN and PA. HG and KSN performed statistical analyses. HG prepared the first draft of the manuscript. All authors contributed to the interpretation of the study findings, revised and approved the manuscript.
